# Testing the Influence of Metakaolinite and Zeolite on the Adhesion of BFRP and GFRP Bars to Concrete

**DOI:** 10.3390/ma16237435

**Published:** 2023-11-29

**Authors:** Julita Krassowska, Paweł Wolka, Kostiantyn Protchenko, Alejandra-Barriguete Vidales

**Affiliations:** 1 Department of Building Structures, Bialystok University of Technology, 15-351 Bialystok, Poland; 2 Astra Technologia Betonu sp. z o.o., 83-010 Straszyn, Poland; pawel@astra-polska.com; 3 Faculty of Civil Engineering, Warsaw University of Technology, Al. Armii Ludowej 16, 00-637 Warsaw, Poland; k.protchenko@il.pw.edu.pl; 4 Departamento de Tencología de la Edificación, Escuela Técnica Superior de Edificación, Universidad Politécnica de Madrid (UPM), Avda. Juan Herrera 6, 28040 Madrid, Spain; alejandra.vidales@upm.es

**Keywords:** basalt fiber-reinforced polymer, basalt fibers, basalt fiber-reinforced polymer stirrups, flexural capacity

## Abstract

Today’s sustainable development policy in Europe, which is driven by concerns about the greenhouse effect and environmental protection, mandates a reduction in CO_2_ emissions into the atmosphere. The cement industry and steel mills that produce reinforcing bars are among the largest and most emissions-intensive sectors emitting CO_2_ into the atmosphere. This article analyzes the possibility of achieving significant reductions in CO_2_ emissions by using basalt bars (BFRP) and glass bars (GFRP) in concrete structures, and—in the case of concrete—by using cement with the addition of metakaolinite and zeolite. There is a lack of literature reports on whether modifying concrete with the additions of metakaolinite and zeolite as substitutes for part of the cement affects the adhesion of FRP bars to concrete. It can be assumed, however, that improving the microstructure of concrete also improves the contact zone between the bar and the concrete. The aim of this research is to fill the aforementioned gap in the literature data by determining how the presence of metakaolinite and zeolite affects the adhesion of reinforcing bars to concrete and testing selected properties of hardened concrete. The test samples were prepared following the appropriate beam test procedure. The obtained results made it possible to perform a comparative analysis of reference samples and those with metakaolinite and zeolite additions. The research showed that introducing active pozzolanic additives in the form of metakaolinite and zeolite into concrete improved adhesion stress values by approximately 20% for glass GFRP bars and 15% for basalt BFRP bars, especially in the destruction phase.

## 1. Introduction

The construction materials market is characterized by a growing interest in the production, distribution, and application of composite rods as reinforcement in concrete elements. Their popularity has been increasing due to their numerous advantages, i.e., corrosion resistance, electrical non-conductivity, insensitivity to electromagnetic fields, and the relatively low weight of the rods. However, it should be noted that the low longitudinal elasticity coefficient and the absence of plastic properties limit their structural applications [1]. Various types of surface finishes are used in the production of FRP rods, such as a wrap of resin-impregnated fiber strips, a quartz sprinkle, or texture applied during production. Nevertheless, the production methods do not guarantee reliable adhesion of FRP rods to concrete [2,3,4,5,6]. Hence, conducting adhesion tests for each of the cases is necessary.

**Figure 1:**
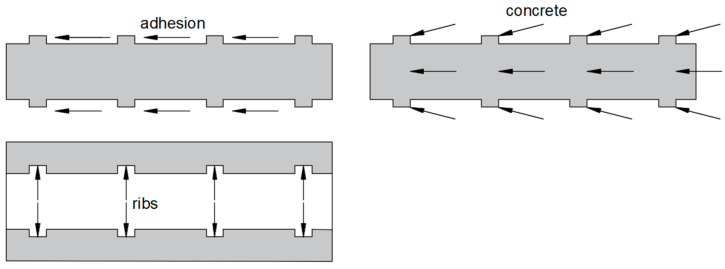
Bond force transfer mechanisms.

A proper interaction between the concrete and the reinforcement enables the transfer of the tensile or compressive stresses that occur in concrete to the reinforcing rods, which form the basis of reinforced concrete. Hosseini [7] identifies the key mechanisms involved in the maintaining of proper adhesion of the rod to the concrete (Figure 1).

The first of these mechanisms is friction, which is particularly significant in the transfer of forces between the bar and the concrete and depends mainly on the ribbing of the bar as the concrete mix can penetrate between the ribs and become wedged. This is related to another mechanism, i.e., mechanical interlocking. The final mechanism is chemical adhesion, i.e., chemical bonding. It generates shear strength in the steel-concrete connection, which can be broken even with minimal relative slip of the bar against the concrete. In addition to slip, adhesion can be compromised due to the formation of small cracks on the surface of the surrounding concrete. The complexity of the issue makes it impossible to unequivocally determine adhesion stresses, which are the result of the various factors described above [8].

Cooperation is primarily possible due to the phenomenon of adhesion that occurs between the materials in question, influenced by factors such as:

- Concrete class (higher concrete classes result in greater adhesion).
- Friction between materials due to their interlocking.
- The surface condition of the reinforcement (a light, non-peeling rust layer increases adhesion).
- The type of reinforcing bars—ribbed bars exhibit 1.5 times greater adhesion than smooth bars.
- Concrete age (over time, concrete strength increases, affecting adhesion).
- Material adhesion—intermolecular attraction.
- Shear of concrete between the concrete ribs.
- Diameter, shape, as well as the arrangement and termination of the reinforcement.
- Placement and consolidation of the concrete mix (vibrating the concrete ensures thorough encasement of the reinforcing bars in the concrete mix).
- Proper curing of fresh concrete.

At each segment of the reinforcing bar, longitudinal forces are balanced by tangential forces due to interactions with the concrete. These are adhesion forces or stresses, which are the greatest at the ends of the bars and occur as a result of the rapid changes in normal stresses along the length of the bar. In the case of long bars arranged in an uncracked tensile section, constant adhesion stress forces equal zero. On the other hand, significantly higher stresses occur in the reinforcement in cracks, compared to uncracked sections. Consequently, every crack induces adhesion forces.

In the case of smooth steel, adhesion is provided by micron-sized irregularities present on the surface of the reinforcement, as well as by the cohesion and shrinkage of concrete. Unlike ribbed steel, smooth steel has a significantly lower yield strength. In ribbed steel, both adhesion and the resistance associated with the phenomenon of reinforcement slippage are provided by the bars’ ribs. Moreover, the adhesion of ribbed reinforcing steel also depends on the size and shape of the ribs present on the surface of the bars [9]. 

Many studies have explored the way in which adhesive stress is transferred between FRP reinforcement and concrete [10,11,12,13,14,15,16]. They focused on analyzing the influence of various factors, such as the type of reinforcement, the length of the embedment, and the cross-sectional shape, with the results presented in publications. Their conclusions indicate that the pullout mechanism for many existing types of FRP reinforcement differs from that observed for steel bars and was dependent on numerous other factors. In the case of smooth-surfaced rebars, it appears that the influence of the concrete’s mechanical properties is minimal, and the adhesive behavior is primarily dependent on the type of fibers and the matrix [17,18]. Overall, a clear general trend emerges in the findings of previous studies, i.e., larger rebar diameters often correspond to lower adhesive strengths [10,13,19].

Numerous studies have been conducted to examine the interaction between FRP reinforcement and concrete, primarily using the direct pull-out test, the beam test, the splice test, or the ring pullout test. However, it is worth noting that the setups for the direct and ring pullout tests do not accurately replicate the actual bonding conditions in a reinforced concrete structure. Therefore, the most accurate assessment of the bonding behavior of reinforcement can be achieved through either the beam test or the splice test.

Moreover, all previous studies demonstrate lower values of bond stresses in the case of FRP bars. For this reason, there is an urgent need to explore ways in which it would be possible to increase their bond strength.

A relatively simple method for improving the adhesion of composite bars to concrete is the modification of the reinforcement-matrix cement contact zone by using active additives in the concrete. This enhances compressive and tensile strength of the concrete, friction between the materials, and material adhesion. What is more, the active additive can also partially replace cement.

One of the valued additives with pozzolanic properties is metakaolin. It is produced from natural kaolin clay in the calcination process at temperatures ranging from 700 to 900 °C [10]. The production of metakaolin is a much less energy-intensive process compared to cement manufacturing as both the temperature and the duration of calcination are reduced. According to [20], producing one ton of metakaolin requires about 80% less energy than producing one ton of cement. Previous studies have shown that metakaolin can improve the mechanical properties and the chemical resistance of concrete [21,22,23,24]. Moreover, an enhanced microstructure of concrete is believed to increase its durability [24,25].

Zeolites are porous silicate minerals containing significant amounts of reactive SiO_2_ and Al_2_O_3_. Preliminary studies have confirmed the beneficial effect of zeolite used as a modifier on the increase in compressive and flexural strength, but only when the zeolite content is below 15% of the cement mass. Furthermore, the addition of zeolite enhances the durability of conventional concrete, not only by reducing concrete permeability but primarily by improving its resistance to the alkali-aggregate reaction.

There is a lack of literature reports on whether modifying concrete with additions of metakaolin and zeolite as substitutes for part of the cement affects the adhesion of FRP bars to concrete. It can be assumed that by improving the microstructure of concrete, the properties of the contact zone between the bar and the concrete will also improve. This study contributes to filling this gap in knowledge.

The aim of this research was to determine how the presence of active pozzolanic additives affects the adhesion of steel, basalt, and glass bars to concrete, as well as its influence on selected properties of the tested concrete.

## 2. Materials and Methods

In the study, both cement concrete and concrete in which 10% of the cement mass was replaced with either metakaolin or clinoptilolite zeolite were used. As reported by de Silva and Glosser, concrete strength is closely related to the reduction in microporosity caused by the presence of metakaolin and zeolite [26]. Essentially, factors such as the amount of the additive have an impact on this parameter. In most cited studies, it typically ranged from 5 to 25%. Another crucial factor affecting the strength of cement paste is the composition of Portland cement to which it was added. When studying the properties of hardened cement paste with the addition of metakaolin, Ambroise et al. found the highest increase in strength when 10% of the cement was replaced with metakaolin [27]. Similarly, Batias et al. state that from the point of view of strength properties, the optimal amount of metakaolin is 15%, but after adding it in the amount of 10%, better properties and greater regularity in the variation of mortar parameters were achieved [28]. 

The influence of the additive on the adhesion of steel, basalt, and glass bars to the surrounding concrete was analyzed. The study was conducted in nine series, as presented in Table 1.

### 2.1. Materials

The composition of the concrete mixture is presented in Table 2.

**Figure 2:**
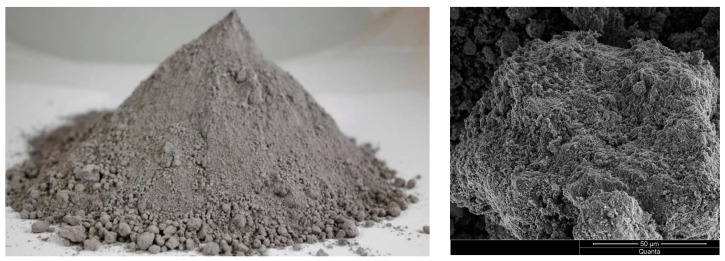
Metakaolin Astra Metakaolinit-40.

ASTRA METAKAOLINIT-40, a highly reactive pozzolanic additive (Figure 2) obtained through the calcination process of kaolinite followed by grinding, was used in this study. ASTRA METAKAOLINIT-40 contains active forms of aluminum and silicon oxides, which readily react chemically with free calcium hydroxide (Ca(OH)_2_). As a result, products in the form of hydrated calcium silicates are formed, which are compositionally and structurally similar to the products of Portland cement hydration. The material has a density of 2.61 g/cm^3^, and its specific surface area, determined using the BET method, is 14.6 m^2^/g. The pozzolanic activity index, tested after 28 days pursuant to EN 450-1:2012 [29] was 115.7%; after 90 days, it was 114.3%.

**Figure 3:**
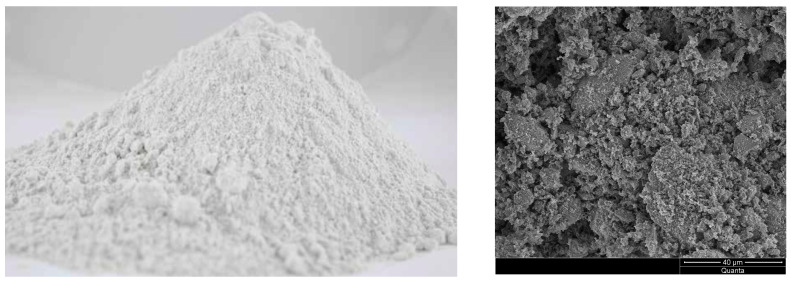
Natural zeolite ASTRA Z-50.

Natural zeolite obtained from the Astra company was used in the study. ASTRA Z-50 (Figure 3) is a natural additive characterized by a fine-grained structure with a specific surface area of 32.5 m^2^/g and a density of 2.29 g/cm^3^, as determined by the BET test. It is obtained in the process of drying and grinding of natural zeolite rock. Its primary component is hydrated alkali aluminosilicate, belonging to the group of clinoptilolite zeolites. Due to its pozzolanic properties and ion exchange capabilities, ASTRA Z-50 has a positive impact on various parameters of concrete mixtures and hardened concrete, such as concrete mixture’s rheology, mechanical strength, impermeability, and resistance to biological, chemical, and environmental aggression [28]. The pozzolanic activity index, tested after 28 days pursuant to EN 450-1:2012 [29] was 102.7%; after 90 days, it was 106.4%.

The reference mixture was designed in accordance with the requirements of the EN 206:2014 standard [30]. The composition of the mineral mixtures was determined using the method of optimal grain size boundary curves. Additionally, the selection of the quantity of the concrete mix components was performed using an analytical–experimental method pursuant to the EN 206:2014 [30] standard.

### 2.2. Methods

The slump [31] and air content [32] were determined in a fresh concrete mixture. Tests of compressive strength *f_ck_* of the concrete were carried out pursuant to EN 12390-3:2011 [33] using cubic samples with a side of 100 mm. Flexural strength *f_ctm_* of the concrete was tested on samples with dimensions of 100 × 100 × 400 mm pursuant to EN 12390-5:2011 [34]. Elasticity modulus *E_cm_* was determined pursuant to EN 12390-13:2014 [35] using cylindrical specimens with a diameter of 150 mm and a height of 300 mm. 

To determine the strength characteristics of non-metallic bars, a batch of steel, basalt fiber-reinforced polymer (BFRP), and glass fiber-reinforced polymer (GFRP) bars were subjected to tensile testing. Before each test, the diameter of the bar was measured at three points along the sample length. A universal testing machine equipped with special inserts for hydraulic grips was used for the axial tensile test, allowing for quick strain control and load adjustment. The measurement results were recorded automatically. The bar samples were loaded at a constant rate to ensure that the test duration fell within the range of 1 to 10 min, in accordance with the ACI 440.3R recommendations [36]. Extensometers were used to determine deformations of the tested samples.

**Figure 4:**
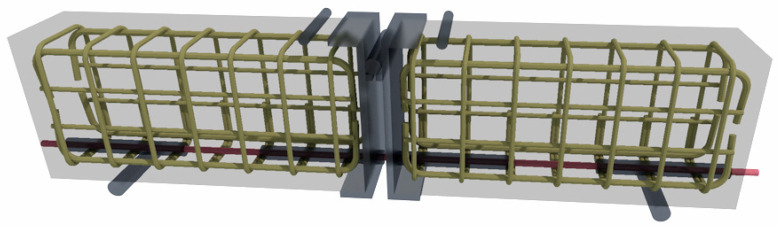
Reinforcement of beams.

To determine the adhesion of steel, basalt, and glass bars to concrete, the pull-out test was used (Figure 4). The test was conducted following the recommendations of standard [37].

Two reinforced concrete beams were connected by a bar placed at the lower part of the element. Adhesion was determined in the middle of each beam. To exclude the adhesion of the bar along the remaining length, plastic conduits were put in place. The sample was positioned on supports in such a way that forces were distributed symmetrically and the proper distance between the supports was maintained. The test continued until there was a complete loss of adhesion by the bar in both beams or until the beam was pulled apart.

**Figure 5:**
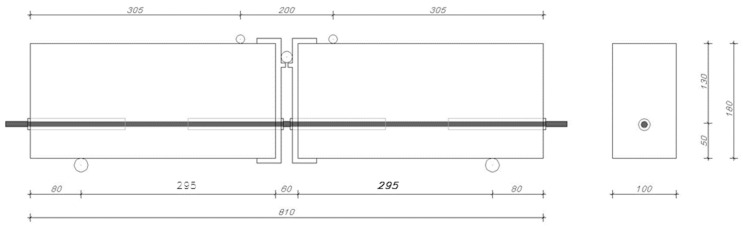
Test set up.

The test set up consisted of two beams, each measuring 100 mm × 180 mm × 375 mm. The adhesion testing was performed on steel-, basalt-, and glass-reinforced bars with a diameter of ϕ8. Additionally, auxiliary reinforcement in the form of a mesh made of ϕ6 steel bars was used in both beams of each of the test specimens (Figure 5). PVC pipes were attached in such a way as to prevent their displacement during concrete pouring or compaction.

The testing apparatus for the pullout test included two rotating roller bearings positioned 650 mm apart from each other. The sample was subjected to two-point loads applied symmetrically in relation to the center of span of the beam with a total length of 810 mm. During the test, the role of the steel joint was to transfer stresses from the compression zone, enabling deflection without causing stresses that would crush the concrete. During the test, the bar in the tensile zone was pulled out of the concrete. Measurements of the force–slip relationship of the bar were taken using sensors with a reading accuracy of 0.001 mm placed at the ends of the bar. To increase stresses σ in the bar, each sample was loaded in the following manner: 0-80-160-240 MPa. In accordance with [37], the total force applied to the tested element for each of the increments was:

(1) $$F_{a}=\frac{A_{n}\times \sigma }{1.25}$$

where $A_{n}$ is the nominal cross-sectional area of the bar [mm^2^]; σ corresponds to the tensile stress in the bar [MPa].

While it is possible to adopt smaller values of stress σ in the bar, it must be ensured that they increase stepwise by a constant amount.

The force increment had to be achieved within 30 s and the load had to be maintained for long enough for the slip to stabilize, but for no longer than 2 min. The force increment, as per the recommendations, was gradual and continuous. The test was conducted until the adhesion of the bar was lost in both beams.

Bond stress $\tau_{b}$, at a given slip value of bar force $F_{a}$, was calculated based on the following formula:

(2) $$\tau_{b}=\frac{\sigma }{4\times 10\times d_{b}}$$

where *σ* is the stress of the tested bar [MPa]; $d_{b}$ is the diameter of the bar [mm].

The bond stress was calculated for the following four measured slip values:

$\tau_{0.01}$—bond stress corresponding to a slip of 0.01 mm.

$\tau_{0.1}$—bond stress corresponding to a slip of 0.1 mm.

$\tau_{1}$—bond stress corresponding to a slip of 1.0 mm.

$\tau_{max}$—bond stress at the maximum pullout force.

## 3. Results

### 3.1. Analysis of Concrete Properties

Table 3 presents the results of the tests of compressive strength ***f_ck_***, tensile strength in bending ***f_ctm_***, and longitudinal modulus of elasticity of concrete ***E_cm_***. For the R series, reference concrete was used; for the M series, concrete with a 10% metakaolin addition was used; for the Z series, concrete with a 10% zeolite addition was used. The average strength was calculated based on the arithmetic mean of individual sample series.

The mixture without the metakaolin additive showed a slightly lower slump height, although the difference was not significant, confirming the same construction class for both samples. The results of the consistency tests of the mixture of concrete with the zeolite additive showed that the mixture with zeolite reduced the flowability of the S4 class mixture (160–210 mm) down to the S1 class values (10–40 mm). This should be taken into account in future studies, i.e., a larger amount of superplasticizer would make it possible to achieve the desired consistency.

Based on the results of the density tests of the concrete mixture, it was found that the density of the concrete with the zeolite additive was 8.22% lower than that of the reference concrete. The lack of a significant change in the composite density was due to the similar densities of metakaolin and sand, with the amount of sand adjusted according to the impermeability condition.

Based on the obtained test results, it was found that concrete with 10% additions of metakaolin and zeolite was characterized by compressive strength values that were comparable to and 20% higher, respectively, than those of the reference concrete. A positive effect of metakaolin and zeolite additives on tensile strength in bending (a 10% increase) and longitudinal modulus of elasticity (a 5% increase) were also observed.

### 3.2. Analysis of the Strength Properties of Bars

For each series, measurements of diameters were taken at three different locations to compare the obtained results with the nominal representative diameter. The results are presented in Table 4. According to Annex C to norm [37], each series included small-diameter bars within the range of d ≤ 10mm.

Compared to steel bars, basalt BFRP bars exhibit 72% greater tensile strength, while glass bars exhibit 45% greater tensile strength. Unlike steel, composite materials do not have a plastic range, i.e., their state preceding failure is not signaled and failure occurs suddenly after the strain limits are exceeded.

### 3.3. Surface Characteristics of FRP Bars

FRP bars are manufactured with various surface deformations to enhance their adhesion to concrete. The FRP bars used in this study were basalt BFRP and glass BFRP, along with reference steel bars. As they all differ in rib geometry, to help interpret the results, the surface characteristics of the bars are analyzed in the subsequent part of the study.

**Figure 6:**
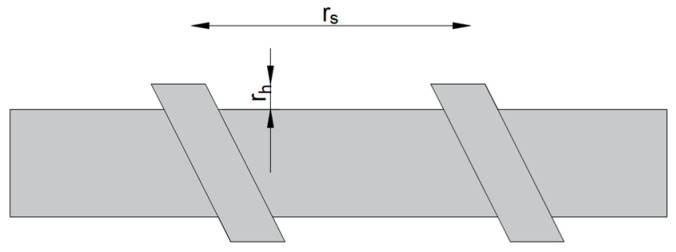
Parameters of FRP bars ($r_{s}$—rib spacing [mm]; $r_{h}$—height of rib [mm]).

To account for the influence of bar geometry on bond behavior, two geometric ratios have been proposed in literature, i.e., a_s_ and R_r_ [16,38,39].

(3) $$a_{s}=\frac{A_{r}}{r_{s}}$$

(4) $$R_{r}=\frac{A_{r}}{Ø\times r_{s}\;}$$

where $a_{s}$—area to space ratio [mm]; $A_{r}$—projected rib area [mm^2^]; $r_{s}$—rib spacing [mm]; *R_r_*—relative rib area [–]; Ø—nominal bar perimeter [mm];

Figure 6 schematically shows the parameters of FRP bars, which are also presented in Table 5.

**Figure 7:**
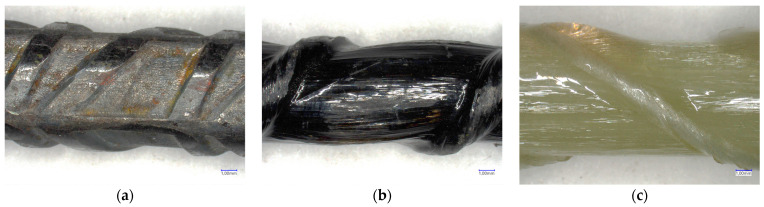
Microscopic image of the tested bars (**a**) steel, (**b**) BFRP, and (**c**) GFRP.

Figure 7 shows example images of the tested steel (a), basalt (b), and glass (c) bars. Surface finish can directly influence the force component of adhesion stress [40]. 

**Figure 8:**
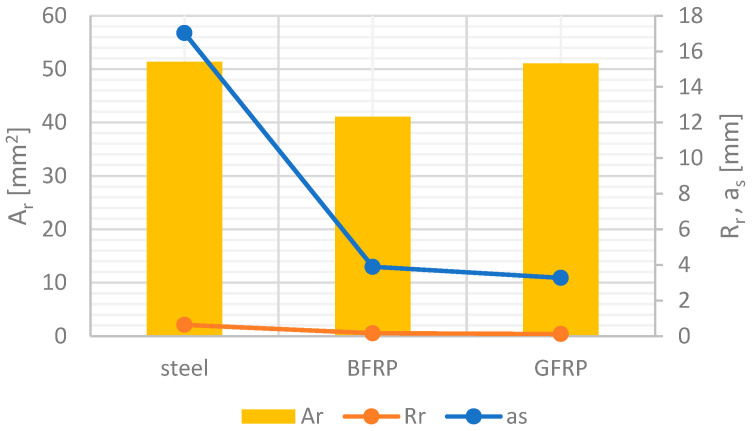
Schematic representation of different deformed bars.

An analysis of Table 5 and Figure 8 shows a clear correlation between them. The values of R_r_ and Ø show a similar trend, while the a_s_ ratio for steel bars is four times higher than that for composite bars. Moreover, the spacing and the sizes of ribs in GFRP bars are the largest, followed by those in basalt bars, with the smallest and least spaced ribs present in steel bars. Steel and glass bars have the highest cross-sectional area values. Therefore, the influence of geometric ratios must be taken into account when analyzing the results obtained for bars produced by different manufacturers or bars of the same type but with different diameters.

**Figure 9:**
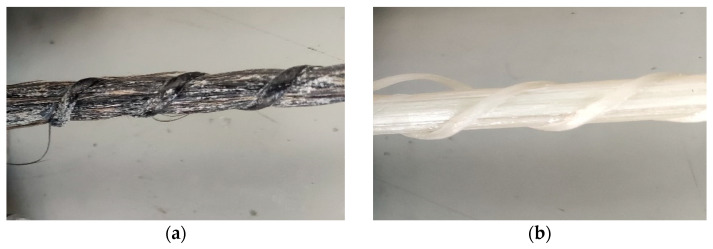
Failure model of FRP bars: (**a**) BFRP and (**b**) GFRP.

In addition to geometric ratios, differences in failure mode should also be considered. The BFRP and GFRP bars used in this study primarily underwent damage through rib debonding, followed by the tearing of individual fibers. The higher the rib of the bar, the higher the bearing forces at rib height, which makes it easier for the rib to detach (Figure 9). Compared to BFRP bars, GFRP bars had ribs that were 10% higher.

### 3.4. Analysis of Tests of Adhesion of Steel and FRP Composite Bars to Concrete

**Figure 10:**
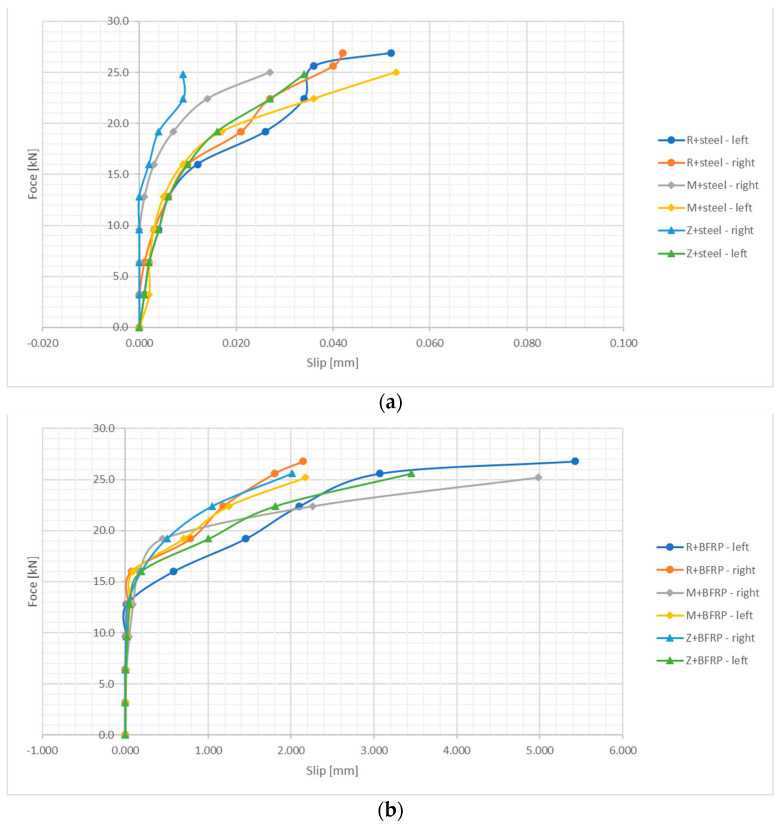

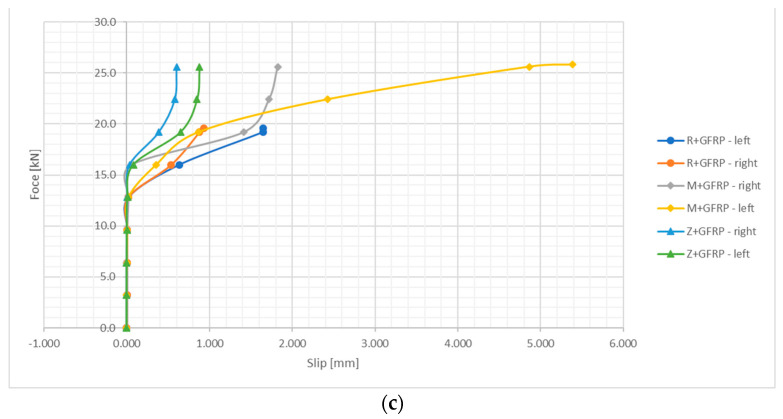
Displacement–force relationship diagram: (**a**) steel bars, (**b**) BFRP bars, (**c**) GFRP bars.

Figure 10 presents the slip values obtained in the tests of bar adhesion to concrete in relation to the applied force. The slip values were higher on the side where adhesion was lost. In the initial phase of loading, a similar trend could be observed on both sensors, which indicates that adhesion and bar slip were equal on both sides of the test element.

Total adhesion failure has a gradual nature, and its mechanism depends on numerous factors. After applying an increasing tensile force to a smooth reinforcing bar embedded in concrete, adhesion—related to both chemical adhesion and friction—is lost at a certain load, resulting in the formation of cracks. This disrupts the continuity of deformations in both materials, which is known as primary adhesion, leading to relative slippage. A further increase in the load promotes the formation of additional radial cracks emanating from the bar and propagating through the concrete cover until its rupture.

In this study, a comparison was made between GFRP and BFRP bars with ribbing in the form of a wrap, and ribbed steel bars of a similar equivalent diameter.

The ultimate criterion for failure in the beam test was rupture of the steel or the composite bars. Complete rupture of the reinforcement in the concrete beams was achieved for none of the tested beams, indicating very good adhesion properties of bars of each of the tested types.

The force–displacement relationship showed a consistent pattern for all the series of steel bars. It was linear in the first phase, while the second phase was characterized by non-linear growth until the maximum adhesive force was reached. Finally, the third phase led to failure. In the series with steel bars, the failure phase was short and sudden. The influence of metakaolin and zeolite as additives to the concrete with steel bars resulted in a slight decrease in the maximum adhesive force (6%).

For basalt BFRP reinforcement, three phases can be distinguished in the force–displacement graphs. The first phase, up to half of the destructive force, is linear, followed by a phase of nonlinear growth, and the third phase of failure. The maximum adhesive force values obtained in the study were 21.9 kN for the reference concrete and 25.4 kN for the concrete with a metakaolin addition. This means that the addition of metakaolin to concrete increases the adhesive force by approx. 6%; the addition of zeolite increases this force by 11%.

The graphs obtained for beams reinforced with GFRP bars show force–displacement characteristics that are similar to the series with BFRP reinforcement. This is due to the material characteristics of these bars, which are similar to basalt BFRP bars. In this case, however, the maximum values of force proved to be the highest for concrete with a metakaolin addition at 25.7 kN and for concrete with a zeolite addition at 26.7 kN.

In the series with composite bars, an increasing incline of the curve can be observed from the second phase onwards, until failure occurs. A plastic plateau and plastic material strengthening were formed, as indicated by the nonlinear character of the graph, which also points to gradual damage at the interface between the concrete and the bar due to mechanical locking and wedging effects.

However, the different behavior of steel bars under load, compared to basalt BFRP and glass GFRP bars, must be confirmed unequivocally. In the conducted tests, it could be observed that composite bars, due to their different types of wrap and their composition consisting of resin and glass, as well as basalt roving, exhibit different mechanical and chemical properties of adhesion between the concrete and the bar. The use of metakaolin resulted in an increase in the compressive and tensile strength of concrete, which is also one of the components responsible for adhesion.

**Figure 11:**
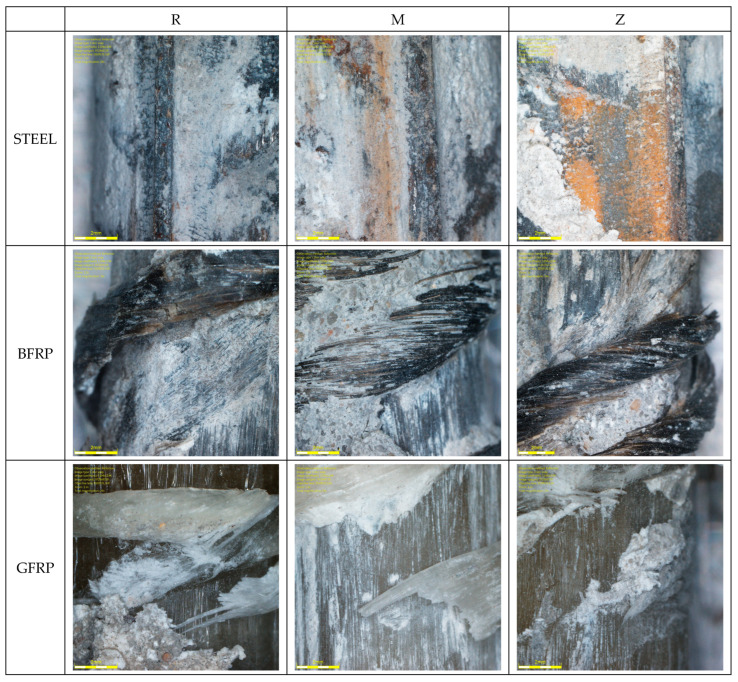
Microscope images of a steel–concrete joint.

Figure 11 presents models of bar damage after adhesion testing. One of the reasons for the lower adhesion of composite bars compared to steel bars is the ribbing used (i.e., ribs made of resin-soaked string) in basalt and glass bars. FRP bars fail mainly due to rib detachment. In this mechanism of force transmission, the smaller the elements that interact with concrete, the better their performance, as upon contact, these elements transfer compressive forces from the concrete to the bar, increasing adhesion. A decrease in the adhesion force values was observed due to material differences, i.e., 9% for basalt BFRP bars and 12% for glass GFRP bars.

Table 6 contains the average values of forces applied to the specimens, determined at slip (displacement of the bar relative to the concrete) values of 0.01 mm, 0.1 mm, and 1.0 mm. The corresponding tensile stresses in composite bars are also presented; these are determined using the formula specified in [35]:

(5) $$\sigma =\frac{1.25\times F_{a}}{A_{p}}10^{3}$$

where: σ—tensile stress in the composite bar [MPa], $F_{a}$—total force applied to the sample [kN], and $A_{p}$—nominal cross-sectional area of the bar [mm^2^].

The results for the tensile stresses in the bar measured at slip show that stresses increase with increasing slippage. In the R-GFRP and M-GFRP series, in which metakaolin was added to the concrete along with glass bars, the highest increase in the values of tensile stresses was observed at all stages, i.e., from 0.01 to failure. In the R-BFRP and M-BFRP series, in which metakaolin was added to the concrete along with basalt bars, a 25% increase in tensile stress was noticed at failure. In the Z series, a remarkable increase in the applied forces at slip, i.e., up to 64%, was observed for glass bars (GFRP). On average, compared to the reference concrete, the increase was approx. 30%.

Basalt bars (BFRP) and glass bars (GFRP) exhibited lower tensile stress values than steel bars. Comparing both types of composite reinforcement used, it can be concluded that glass bars were characterized by higher tensile stress values in the bar.

In the case of BFRP bars, despite the greater spacing in the wrap compared to the ribs in steel bars, the adhesion values were higher than those obtained for steel bars. The loss of adhesion in both GFRP and BFRP bars was caused by complete deformation of the wrap’s ribs. Damage was initiated at the base of the wrap and then propagated outward. Ultimately, in both GFRP and BFRP bars, the shearing surface appeared at the interface between the concrete and the wrap. The adhesion mechanism was primarily enacted by the mechanical interlocking caused by the wrap of the composite bar. Hence, it can be stated that in the case of the adhesion mechanism, in addition to the aforementioned influence of the concrete composition, the type of wrap deformation also plays an equally important role.

Table 6 provides the average adhesion stress values determined for the part of the specimen where adhesion was lost, calculated using the following formula [35]:

(6) $$\tau =\frac{\sigma }{40}$$

where: τ—adhesion stress of the composite bar [MPa], σ—tensile stress in the composite bar [MPa].

According to EN2 annex C [41] and RILEM [42], adhesion stresses should meet the following condition:

(7) $$\tau_{m}=0.098\left(80-1.2Ø\right)$$

(8) $$\tau_{r}=0.098\left(130-1.9Ø\right)$$

where: Ø—nominal diameter of the bar [mm], $\tau_{m}$—average value of adhesion stress [MPa] at slip values of 0.01, 0.1, and 1 mm, $\tau_{r}$—adhesion stress at the moment of failure by slipping.

**Figure 12:**
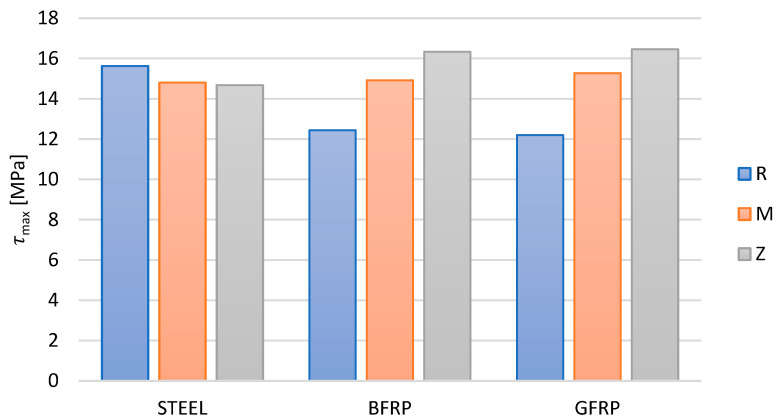
The influence of zeolite and metakaolin additions to concrete on the change in the maximum adhesion stresses of the tested types of bars at $\tau_{max}$.

Compared to steel bars, the average adhesion stresses in all the series with composite reinforcement were characterized by lower adhesion. However, the introduction of an active pozzolanic additive into the concrete, in the form of metakaolin and zeolite, resulted in adhesion stress values improved by approximately 20% for glass bars (GFRP) and approximately 15% for basalt bars (BFRP), especially in the failure phase (Figure 12). In the cases in which concrete with additives was used, the contact zone exhibited a tight and compact structure. As a result, the adhesion of basalt and glass bars proved to be stronger compared to the reference concrete. This is due to the formation of a tight layer that filled the free spaces located at the surface between the aggregate and the concrete, which were too small for the cement slurry to penetrate.

In all the tested series, the loss of adhesion in both steel and composite bars was observed in the final stage of loading. According to the EN2 standard [41] and RILEM [42], i.e., in formulas (3) and (4), respectively, the conditions for minimum adhesion were met in all the tested series. In the composite bars in the failure phase, the adhesion stress values increased by 10% for basalt bars and 7% for glass bars. However, with the use of concrete with a 10% metakaolinite or zeolite addition, this increase was twice as high, reaching 32% for basalt bars and 35% for glass bars.

Compared to the flexural tensile strength of the reference concrete or concrete with metakaolin $f_{ctm}$, the values of adhesion stresses at a slip of 0.01 mm $\tau_{0.01}$ indicated a better adhesion of glass bars (GFRP) compared to basalt bars (BFRP). The addition of metakaolin resulted in increased adhesion stresses in both cases, which was caused by the higher flexural tensile strength of concrete.

**Figure 13:**
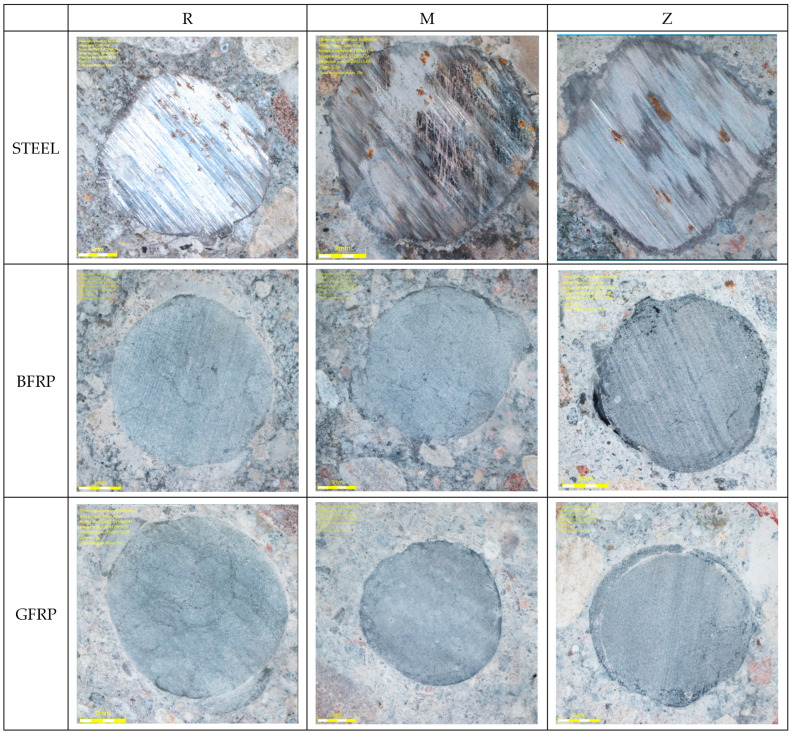
Microscope images of the steel–concrete contact zone.

The diverse adhesion force values inspired the authors to conduct detailed studies of the contact zone between the bars and the concrete with the use of microscopic analysis, as presented in Figure 13.

The microscopic image of the concrete structure presented in Figure 13 reveals the highly complex and porous nature of the material, showing the cement grains that form the matrix of the material. They interconnect, creating a durable binder structure. Moreover, the structure of concrete contains the grains of the aggregates, which are embedded in the cement binder. The concrete does not contain a large number of empty spaces; those that do appear, however, form as a result of the concrete mixing and vibration process, as evident from the images, indicating that the process proceeded correctly.

In the case of concrete with a metakaolin or zeolite addition, the sealing of the concrete structure can be observed (as confirmed by the higher results of the strength test of the concrete). It can be assumed that metakaolin and zeolite act as sealing fillers, reducing the total porosity of the surface, as confirmed in [43]. Frias reports that adding up to 20% of metakaolin to cementitious mortar does not affect the value of total porosity, which ranges from 30% to 34%. However, this does not mean that metakaolin has no effect on the pore structure in the mortar – it does undergo a change, which is best illustrated by the average pore size. With the increase in the metakaolin content from zero to 25%, the average pore size decreases from 34.6 nm to approximately 13 nm, representing a 63% reduction. Only the addition of 25% of calcined kaolinite results in reduced values of total porosity, down to approx. 29%.

## 4. Conclusions

The aim of this study was to perform comparative research on the adhesion of steel, basalt, and glass bars to reference concrete and to concrete with active pozzolanic additives in the form of metakaolin or zeolite. The strength of the concrete was tested and the mechanical properties of steel and of the composite bars were analyzed. Based on the obtained results, it can be concluded that:

- The addition of metakaolin and zeolite to concrete had a positive effect on the adhesion of reinforcing bars to concrete;
- The force–displacement relationship showed a consistent pattern in all the tested series;
- The maximum force values achieved during the tests of the adhesion of basalt bars to concrete were 21.9 kN for reference concrete, 25.4 kN for concrete with metakaolin, and 26.7 kN for concrete with zeolite;
- The GFRP bars in beams exhibited similar force–displacement curves to the basalt reinforcement series, but the maximum force value was the highest, reaching 26.7 kN;
- The use of metakaolin improved adhesion by 8% for basalt BFRP bars and 28% for glass GFRP bars;
- The use of zeolite improved adhesion by 11% for basalt BFRP bars and 33% for glass GFRP bars;
- The addition of active pozzolanic additives in the form of metakaolin to concrete improved the adhesion stress values ($\tau_{max}$) by approximately 20% for glass bars and 15% for basalt bars, especially in the destruction phase;
- Adhesion stresses at slip met the normative conditions in all the series.

The limitations of this research and the methodologies apply only to the two specific types of composite bars.

In future research, the authors plan to conduct detailed SEM analyses of the contact zone between the FRP bar and concrete. This will help explain the beneficial influence of active pozzolanic additives. Furthermore, crucial considerations will also focus on a numerical analyses of bond zones, which will make it possible to design FRP structures with metakaolin and zeolite without the need for additional scientific research.

## Figures and Tables

**Table 1:** The research program (type of concrete: R—reference, M—metakaolin, Z—zeolite; type of bars: steel, BFRP—basalt fiber-reinforced bars, GFRP—glass fiber-reinforced bars).

	Reference Concrete	Concrete with Metakaolin (10%)	Concrete with Zeolite (10%)
Steel	R+steel-01	M+steel-01	Z+steel-01
	R+steel-02	M+steel-02	Z+steel-02
	R+steel-03	M+steel-03	Z+steel-03
BFRP	R+BFRP-01	M+BFRP-01	Z+BFRP-01
	R+BFRP-02	M+BFRP-02	Z+BFRP-02
	R+BFRP-03	M+BFRP-03	Z+BFRP-03
GFRP	R+GFRP-01	M+GFRP-01	Z+GFRP-01
	R+GFRP-02	M+GFRP-02	Z+GFRP-02
	R+GFRP-03	M+GFRP-03	Z+GFRP-03

**Table 2:** Mixture proportions for the concrete.

Mixture Proportions	Quantity
Cement 42.5R, kg/m^3^	360
Water, kg/m^3^	162
Sand 0.125–4 mm, kg/m^3^	709
Aggregate 2/8, kg/m^3^	709
Aggregate 8/16, kg/m^3^	631
Metakaolin or Zeolit	36

**Table 3:** Measured concrete parameters (compressive strength *f_ck_*, tensile strength in bending *f_ctm_*, and longitudinal modulus of elasticity of concrete *E_cm_*).

Concrete	Slump	Density	*f_ck_*	*f_ctm_*	*E_cm_*
	mm	kg/m^3^	MPa	MPa	GPa
R	200 ± 2.35	2529.5 ± 3.25	51.96 ± 1.57	3.55 ± 0.27	29.33 ± 1.01
M	210 ± 1.63	2390.5 ± 0.36	62.15 ± 0.87	3.84 ± 0.65	30.55 ± 1.35
Z	35 ± 1.17	2321.5 ± 0.87	59.33 ± 6.59	3.79 ± 0.10	33.25 ± 0.98

**Table 4:** Measured bar parameters (*Ø*—diameter, *f_u_*—tensile strength, *A_gt_*—elongation, *E*—modulus of elasticity).

Bars	*Ø*	*f_u_*	*A_gt_*	*E*
	mm	MPa	%	GPa
STEEL	8.09 ± 1.60	511.2 ± 2.8	8.2 ± 1.3	215.8 ± 1.3
BFRP	7.24 ± 1.58	879.5 ± 18.3	4.2 ± 4.0	39.1 ± 2.4
GFRP	8.06 ± 2.56	744.3 ± 43.7	2.45 ± 2.4	37.81 ± 2.6

**Table 5:** Surface characteristics of FRP bars.

Bars	$r_{s}$	$r_{h}$	Ø	$A_{r}$	$a_{s}$	$R_{r}$
	Mm	mm	mm	mm^2^	Mm	-
Steel	3.02	0.70	8.09	51.4	17.03	2.11
BFRP	10.56	1.06	7.24	41.1	3.89	0.54
GFRP	15.58	1.16	8.06	51.0	3.28	0.41

**Table 6:** Results of tensile stress measurements in the bar.

Bars	Average Value of Force Applied to the Sample [kN] at Slip				Average Tensile Stresses [MPa] at Slip			
	*F*_0.001_	*F*_0.1_	*F*_1_	*F_max_*	$\sigma_{0.01}$	$\sigma_{0.1}$	$\sigma_{1}$	$\sigma_{max}$
	kN	kN	kN	kN	MPa	MPa	MPa	MPa
R-steel	12.8	26.4	26.4	26.4	303.1	625.2	625.2	625.2
R-BFRP	12.1	19.2	19.2	21.0	286.6	454.7	454.7	497.3
R-GFRP	11.7	16.0	16.0	20.6	277.1	378.9	378.9	487.9
M-steel	13.4	22.4	24.0	25.0	316.9	530.5	568.4	592.1
M-BFRP	11.8	12.8	16.0	25.2	279.0	303.1	378.9	596.8
M-GFRP	12.5	19.2	19.2	25.8	296.0	454.7	454.7	611.0
Z-steel	16.0	22.4	19.2	24.8	378.9	530.5	454.7	587.3
Z-BFRP	16.0	16.0	19.2	27.6	378.9	378.9	454.7	653.7
Z-GFRP	19.2	16.0	19.2	27.8	454.7	378.9	454.7	658.4

## Data Availability

All data used to support the study are included within the article.
